# Integrative genomic analysis reveals functional diversification of APOBEC gene family in breast cancer

**DOI:** 10.1186/s40246-015-0056-9

**Published:** 2015-12-18

**Authors:** Yanfeng Zhang, Ryan Delahanty, Xingyi Guo, Wei Zheng, Jirong Long

**Affiliations:** Division of Epidemiology, Department of Medicine, Vanderbilt University Medical Center, Nashville, 37203 USA; Present address: HudsonAlpha Institute for Biotechnology, Huntsville, 35806 USA

**Keywords:** APOBEC family, Estrogen receptor, Breast cancer, Methylation, Mutagenesis, Chromatin state

## Abstract

**Background:**

The human APOBEC protein family plays critical but distinct roles in host defense. Recent studies revealed that APOBECs mediate C-to-T mutagenesis in multiple cancers, including breast cancer. It is still unclear whether APOBEC gene family shows functional diversification involved in cancer mutagenesis.

**Results:**

We performed an integrated analysis to characterize the functional diversification of APOBEC gene family associated with breast cancer mutagenesis relative to estrogen receptor (ER) status. Among the APOBEC family, we found that both *APOBEC3B* and *APOBEC3C* mRNA levels were significantly higher in estrogen receptor negative (ER−) subtype compared with estrogen receptor positive (ER+) subtype (*P* < 2.2 × 10^−16^ and *P* < 3.1 × 10^−5^, respectively). Epigenomic data further reflected the distinct chromatin states of *APOBEC3B* and *APOBEC3C* relative to ER status. Notably, we observed the significantly positive correlation between the APOBEC3B-mediated mutagenesis and *APOBEC3B* expression levels in ER+ cancers but not in ER− cancers. In contrast, we discovered the negative correlation of *APOBEC3C* mRNA levels with base-substitution mutations in ER− tumors. Meanwhile, we observed that breast cancers in carriers of germline deletion of *APOBEC3B* gene harbor similar mutation patterns, but higher mutation rates in the T*C*W motif (W corresponds to A or T) than cancers in non-carriers, indicating additional factors may also induce carcinogenic mutagenesis.

**Conclusions:**

These results suggest that functional potential of APOBEC3B and APOBEC3C involved in cancer mutagenesis is associated with ER status.

**Electronic supplementary material:**

The online version of this article (doi:10.1186/s40246-015-0056-9) contains supplementary material, which is available to authorized users.

## Background

In the human genome, APOBEC family comprises of a total of 11 genes. They have been known to encode Zn^2+^-dependent DNA cytosine deaminases. Of these 11 members, seven apolipoprotein B mRNA editing enzyme, catalytic polypeptide-like 3 genes (*APOBEC3A*, *APOBEC3B*, *APOBEC3C*, *APOBEC3D*, *APOBEC3F*, *APOBEC3G*, and *APOBEC3H*) are tandemly distributed on chromosome 22 [1]. The other four members are located on other chromosomes, including *APOBEC1*, *APOBEC2*, *APOBEC4*, and activation-induced cytidine deaminase (*AICDA*). The APOBEC family proteins show different classes of DNA cytosine deaminase domains and distinct tissue expression profiles [2–5], indicating they may have diverse biological functions. For example, the APOBEC proteins exhibit different activities in restricting virus replication and inhibiting LINE-1 retrotransposition [6–8].

Recent studies showed the elevated expression of *APOBEC3B* in multiple tumors [9–12]. Meanwhile, the strong correlation of somatic base-substitution mutation with *APOBEC3B* mRNA levels in cancer samples has implicated APOBEC3B as an enzymatic source inducing the C-to-T somatic mutations [9, 10, 13, 14]. However, in breast cancer subtypes, the distinct mutational patterns and genomic changes have also been reported [15–18], meaning that the APOBEC-mediated mutagenesis and APOBEC family expression may be variable in cancer subtypes [14].

Thanks to two projects, The Cancer Genome Atlas (TCGA) [17] and the Encyclopedia of DNA Elements (ENCODE) project [19], leveraging these diverse types of data to interpret functional features of genes or gene families in particular cancer types is possible. Here, we performed integrated analysis on diverse high-throughput sequencing data involved in somatic mutation, gene expression, and epigenetic profiles. The aim of this study is to investigate the functional diversification of the APOBEC family genes in breast cancer, in cancer subtypes with a focus on estrogen receptor (ER) status.

## Results

### Data summary

For breast cell lines, a total of 42 high-throughput sequencing data, including 10 RNA sequencing (RNA-seq) data from two data sets [20, 21], 18 ChIP sequencing (ChIP-seq) data, 4 corresponding input DNA (control) from five data sets, and 10 bisulfite sequencing (BS-seq) data from two data sets [19–22], were collected in this study (Additional file 1: Table S1). Among ten RNA-seq data, eight are from breast cancer cell lines and two are from normal breast cell lines.

Of the 18 ChIP-seq datasets, six histone modifications, including histone H3 lysine 4 methylations (H3K4me1, H3K4me3), lysine 9 trimethylation (H3K9me3), lysine 27 trimethylation (H3K27me3), lysine 36 trimethylation (H3K36me3), and histone H3 lysine 27 acetylations (H3K27ac), were conducted in each of the HCC1954 cell line, an estrogen receptor negative (ER−) breast cancer cell line, MCF-7 cell line, an estrogen receptor positive (ER+) breast cancer cell line, and human mammary epithelial cells (HMEC), a normal breast cell line.

For the ten bisulfite-seq datasets, two included whole-genome BS-seq at the single nucleotide resolution in the ER− breast cancer cell line HCC1954 and the normal cell line HMEC [20]. Eight were generated by reduced representation bisulfite sequencing (RRBS) in low depth [21]. As no or low sequencing coverage in the proximal promoters of APOBECs in the initial analysis, we discarded these eight RRBS data for further analyses.

RNA-seq data was available for 1000 breast tissue specimens from TCGA database, including 915 breast carcinoma samples and 85 adjacent normal breast tissues (Table 1). Among 915 breast tumor samples, 664, 196, and 55 are from breast tumor patients with ER+, ER−, and unknown ER status, respectively.

**Table 1:** Summary of breast cell lines and tissue specimens in the study

	Breast non-tumor cell lines	Breast cancer cell lines	Breast tumor tissues	Adjacent normal breast tissues
No. of samples	2	8	915	85
ER status				
ER+	0	4 (50 %)	664 (72.6 %)	67 (78.9 %)
ER−	2 (100 %)	4 (50 %)	196 (21.4 %)	15 (17.6 %)
Unknown	0	0	55 (6.0 %)	3 (3.5 %)
CNV				
CN0	na	na	28 (3.1 %)	2 (2.4 %)
CN1	na	na	162 (17.7 %)	17 (20 %)
CN2	na	na	597 (65.2 %)	66 (77.6 %)
Unknown	na	na	128 (14.0 %)	0
Availablity of exome-seq data				
Yes	na	na	750 (82.0 %)	na
No	na	na	165 (18.0 %)	na

### Expression profiling of APOBECs in breast cancer

**Fig. 1:**
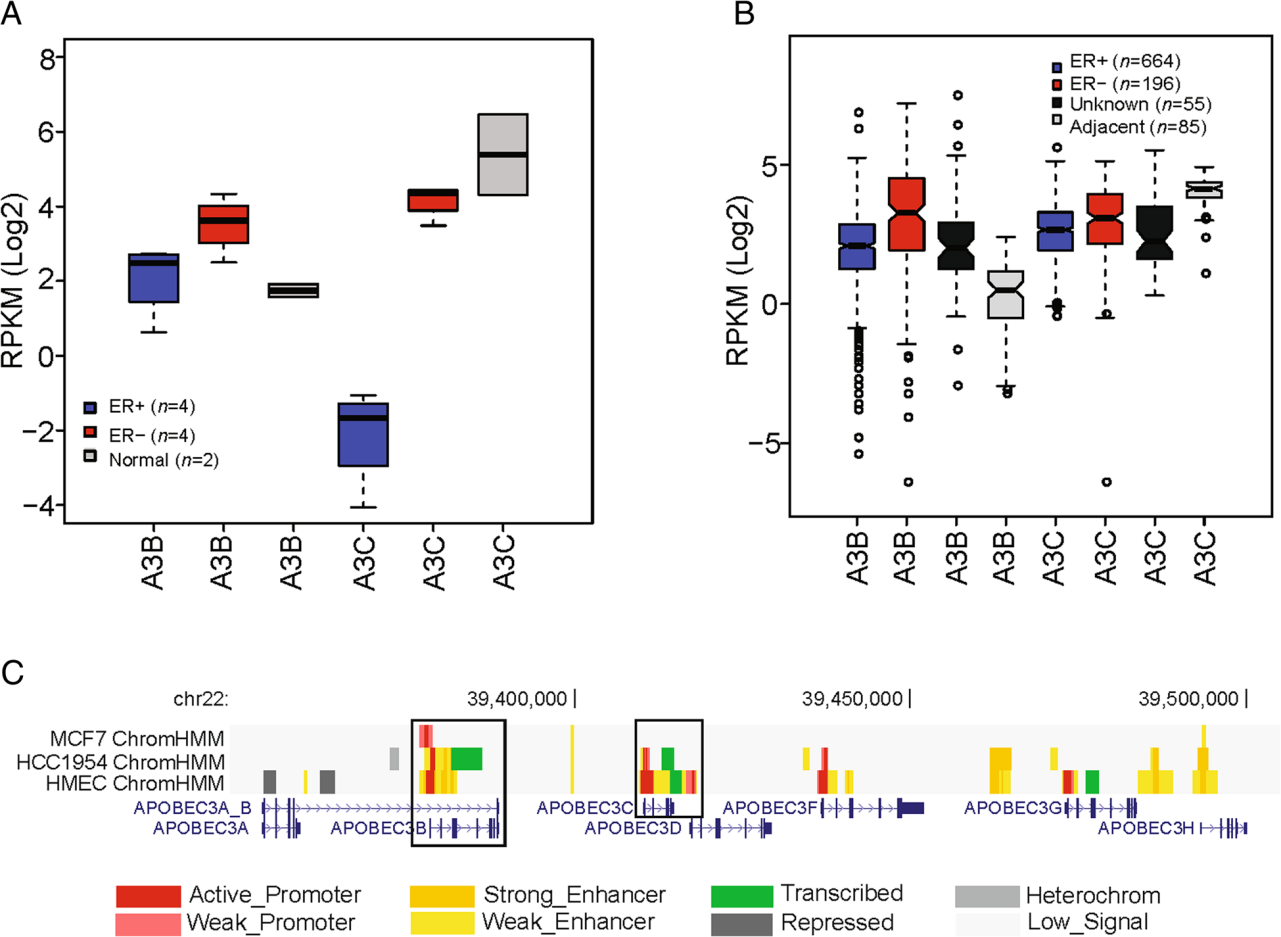
Expression of *APOBEC3B* and *APOBEC3C* genes in breast cancer cell lines and tissues in relation to ER status. **a** mRNA levels of *APOBEC3B* and *APOBEC3C* in breast cancer cell lines with ER subtypes and normal cell lines. **b** mRNA levels of *APOBEC3B* and *APOBEC3C* in breast tumor tissues with ER subtypes and adjacent normal tissues. **c** Comparison of chromatin states in APOBEC3 family genes in ER+ (MCF-7), ER− (HCC1954) breast cancer cells, and normal cells (HMEC). The highlight with rectangle is on *APOBEC3B* and *APOBEC3C* genes. Chromatin states characterized by the ChromHMM algorithm are represented by different colors shown in the *bottom*

We quantified mRNA levels for each of the 11 APOBEC family members in ten breast cell lines including cancer and normal cell types (Additional file 1: Table S2). Consistent with the previous findings [9], only *APOBEC3B* shows the over-expression in the range of two- to fourfold changes in breast cancer cells relative to normal cells. Other APOBEC family members have no or very low expression levels in normal or cancer cells with an exception of *APOBEC3C*, whose expression levels are highest in normal cells and decline slightly in the ER− cancer cell lines, but drop sharply in the ER+ cancer cell lines (Fig. 1a). Relative to ER− cancer cell lines, these results show the down-regulation of both the *APOBEC3B* and *APOBEC3C* genes in ER+ cancer cells.

We then expanded this analysis to include a total of 1000 RNA-seq data from TCGA project. In agreement with observation in cancer cell lines, both the *APOBEC3B* and *APOBEC3C* genes show higher expression levels in ER− cancers (*P* < 2.2 × 10^−16^ and *P* < 3.1 × 10^−5^, respectively, two-sided Wilcoxon rank sum test) relative to ER+ cancers (Fig. 1b). For other APOBEC family members (Additional file 2: Figure S1), they show either extremely low (median log2-transformed RPKM < 0) mRNA levels (including *APOBEC3A*, *APOBEC3H*, *APOBEC1*, *APOBEC2*, *APOBEC4*, and *AICDA*), or no obvious expression differences between ER+ and ER− breast cancers (including *APOBEC3D*, *APOBEC3F*, and *APOBEC3G*). Additionally, the expression levels of APOBEC family genes in 55 breast cancer tissues with unknown ER status are similar with those samples of ER+ subtypes, indicating many of these 55 patients probably belong to ER+ subtype. These results together indicate that the expression patterns of *APOBEC3B* and *APOBEC3C* genes may be associated with ER status.

### Chromatin states of APOBEC gene family in breast cancer cells

To investigate whether the different expression profiles of APOBEC family members could show different chromatin states in relation to ER status, we integrated ChIP-seq data for six histone modifications, including H3K4me3 and H3K36me3 (two epigenetic markers connected with gene activity) [23, 24], H3K27ac and H3K4me1 (two enhancer-associated hallmarks) [25, 26], and H3K9me3 and H3K27me3 (two typical epigenetic markers indicative of transcriptional repression and heterochromatin formation) [23, 27], across three breast tissue relevant cell types: ER+ (MCF-7), ER− (HCC1954) breast cancer cells, and normal mammary epithelial cells (HMEC). Notably, in the ER− cell line HCC1954, we observed active promoter and clustered enhancers surrounding both the *APOBEC3B* and *APOBEC3C* loci (Fig. 1c). Meanwhile, these two genes show strong transcription signal. In addition, parts of active promoters or enhancers at the *APOBEC3F* and *APOBEC3G* genes were also found. For the other APOBEC family genes, we did not observe active promoter or enhancers (Additional file 2: Figure S2).

In contrast, in the ER+ cancer cell line MCF-7, we only observed promoter (Fig. 1c) activity at the *APOBEC3B*, but not at the *APOBEC3C* and the other APOBEC family genes (Additional file 2: Figure S2). Furthermore, compared with the normal HMECs, the loss of promoter activity at the *APOBEC3C*, *APOBEC3F*, and *APOBEC3G* genes was specifically detected in the ER+ cancer cell (Fig. 1c). The observed chromatin state profiles of the APOBEC family genes are quite concordant with the gene expression patterns, further suggesting that the functional divergence of the *APOBEC3B* and *APOBEC3C* genes may be related to the ER status.

In addition, with respect to several APOBEC family members (including *APOBEC3A*, *APOBEC3H*, *APOBEC1*, *APOBEC2*, *APOBEC4*, and *AICDA*) showing no or extremely low expression levels in both ER+ and ER− breast cancers, we did not find any enrichment of repressive chromatin modifications (H3K9me3 and H3K27me3) in these genes (Fig. 1c and Additional file 2: Figure S2), suggesting some additional molecular mechanisms probably participate in the regulation of these genes.

### DNA methylation of APOBECs in breast cancer cells

**Fig. 2:**
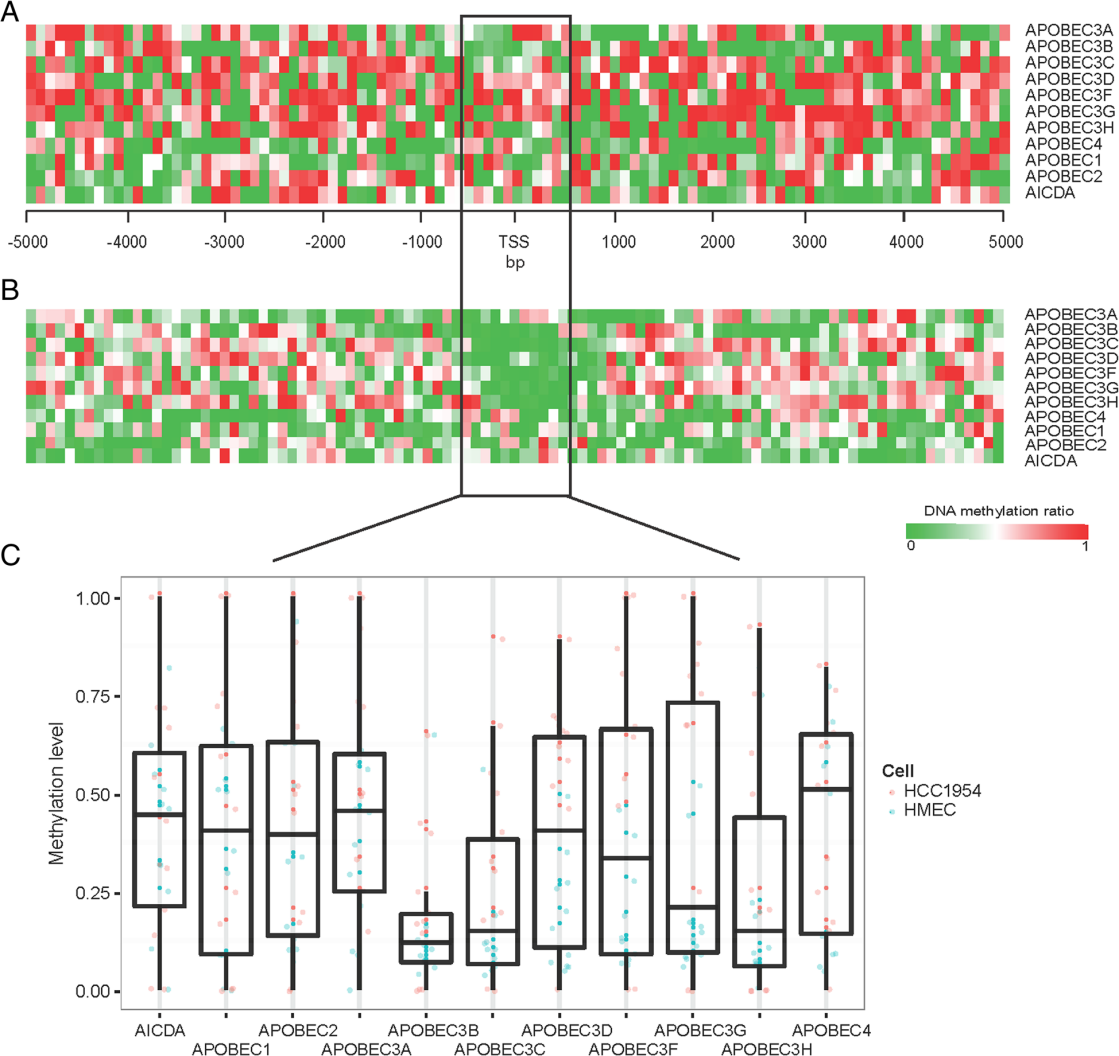
DNA methylation of APOBECs in breast cancer cells. Heatmap of DNA methylation levels per 100 bp bin within ±5 kb regions of APOBEC gene family transcription start sites (TSS) in HCC1954 ER− breast cancer cells (**a**) and HMEC normal breast cells (**b**). The *color bar* shown represents the DNA methylation ratio from the lowest (*green*) to the highest (*red*). **c** Boxplot of DNA methylation levels of APOBECs in breast cancer and normal cells in the proximal regions (defined as ±0.5 kb of TSS)

DNA methylation at the proximal promoter of genes is well-characterized as an epigenetically repressive marker [28, 29]. We further analyzed the DNA methylation profiles of APOBECs using BS-seq data to detect methylation signatures. Due to unavailability of whole-genome BS-seq data for ER+ cancer cells, we could not conduct the comparative DNA methylation analysis between ER+ and ER− cancer cells. Here, we only compared the DNA methylation levels of APOBECs between the ER− breast cancer cells and normal cells (Fig. 2a, b). As expected, highly expressed genes possess low DNA methylation levels in their proximal transcription start site (TSS) regions (defined as ± 0.5 kb of TSS). For example, *APOBEC3B* and *APOBEC3C* show low DNA methylation levels (median methylation ratio ≤20 %) in both HMEC and ER− breast cancer cells, although a slight increase in ER− cancer cells (Fig. 2c). While for other APOBEC members, including *APOBEC3D*, *APOBEC3F*, and *APOBEC3G*, they show DNA hyper-methylation in ER− breast cancer cells when compared to HMEC (*P* < 0.01, Wilcoxon signed rank test). In addition, except the *APOBEC3H* gene, we found high DNA methylation levels of the remaining APOBEC members, including *APOBEC1*, *APOBEC2*, *APOBEC3A*, *APOBEC4*, and *AICDA*, in both HMEC and ER− breast cancer cells (Fig. 2c). Together, these findings are in agreement with the observation from the gene expression data.

### Clinical outcome with mRNA levels of APOBECs

**Fig. 3:**
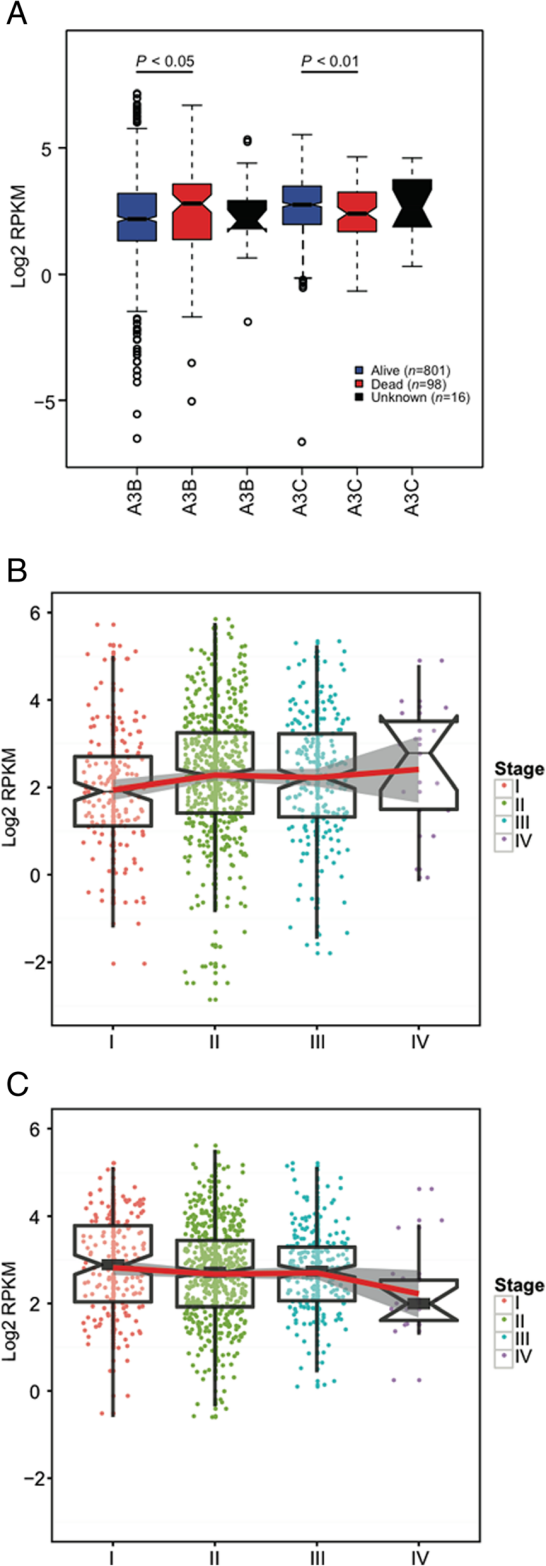
Association of clinical outcome with expression levels of *APOBEC3B* and *APOBEC3C*. **a** Expression levels of *APOBEC3B* and *APOBEC3C* in breast cancer patients who are alive or not. **b** and **c** Association of mRNA levels of *APOBEC3B* and *APOBEC3C* with breast tumor stages ranging from I to IV

We then assessed whether the mRNA levels of both *APOBEC3B* and *APOBEC3C* genes were associated with clinical outcome. Compared to patients who are deceased, surviving patients show lower expression levels of *APOBEC3B* and higher expression levels of *APOBEC3C* (Fig. 3a). Meanwhile, a similar trend was observed for TNM stage, with higher *APOBEC3B* (Fig. 3b) and lower *APOBEC3C* (Fig. 3c) mRNA level associated with advanced breast cancer stage. However, no association was observed between the *APOBEC3B* and *APOBEC3C* mRNA levels with survival times (Additional file 2: Figure S3).

### APOBEC-mediated mutagenesis in relation to ER status

**Fig. 4:**
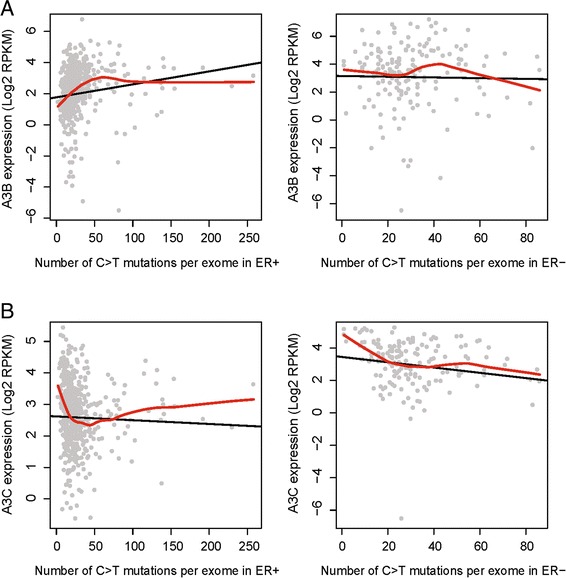
Relationship between mRNA levels of *APOBEC3B* (**a**) and *APOBEC3C* (**b**) and number of C>T/G>A per tumor exome stratified by the ER status. The *black lines* and *red curves* are drawn from the linear regression model and local regression smoothing, respectively

We examined whether the correlation between APOBEC-mediated mutagenesis and expression levels of APOBEC family genes would be associated with ER status. A total of 46,096 somatic single nucleotide variants (SNVs) (57.9 ± 50.9, mean ± SD, Additional file 2: Figure S4A) from 750 exome-sequencing (exome-seq) data and matched RNA-seq data were combined to conduct such test. Among these somatic SNVs, 21,339 are C>T/G>A mutations (27.1 ± 24.8, mean ± SD, Additional file 2: Figure S4B). Interestingly, the significantly positive correlation between the number of C>T/G>A mutations per tumor exome and *APOBEC3B* mRNA levels is observed in ER+ cancers (*ρ* = 0.32, *P* = 7.09 × 10^−15^), but not in ER− cancers (*ρ* = 0.04, *P* = 0.60, Fig. 4a). Locally weighted polynomial regression also shows a similar trend. Conversely, we found a significantly negative correlation between the total number of C>T/G>A mutations per tumor exome and *APOBEC3C* mRNA levels in ER− cancers (*ρ* = −0.26, *P* = 0.001), which is also supported by the locally weighted regression (Fig. 4b). However, the negative correlation for *APOBEC3C* is not obvious in ER+ cancers. Similar observations exist for both *APOBEC3B* and *APOBEC3C* when including all somatic mutations (Additional file 2: Figure S5).

### APOBEC-mediated mutagenesis in relation to germline deletion of *APOBEC3B*

**Fig. 5:**
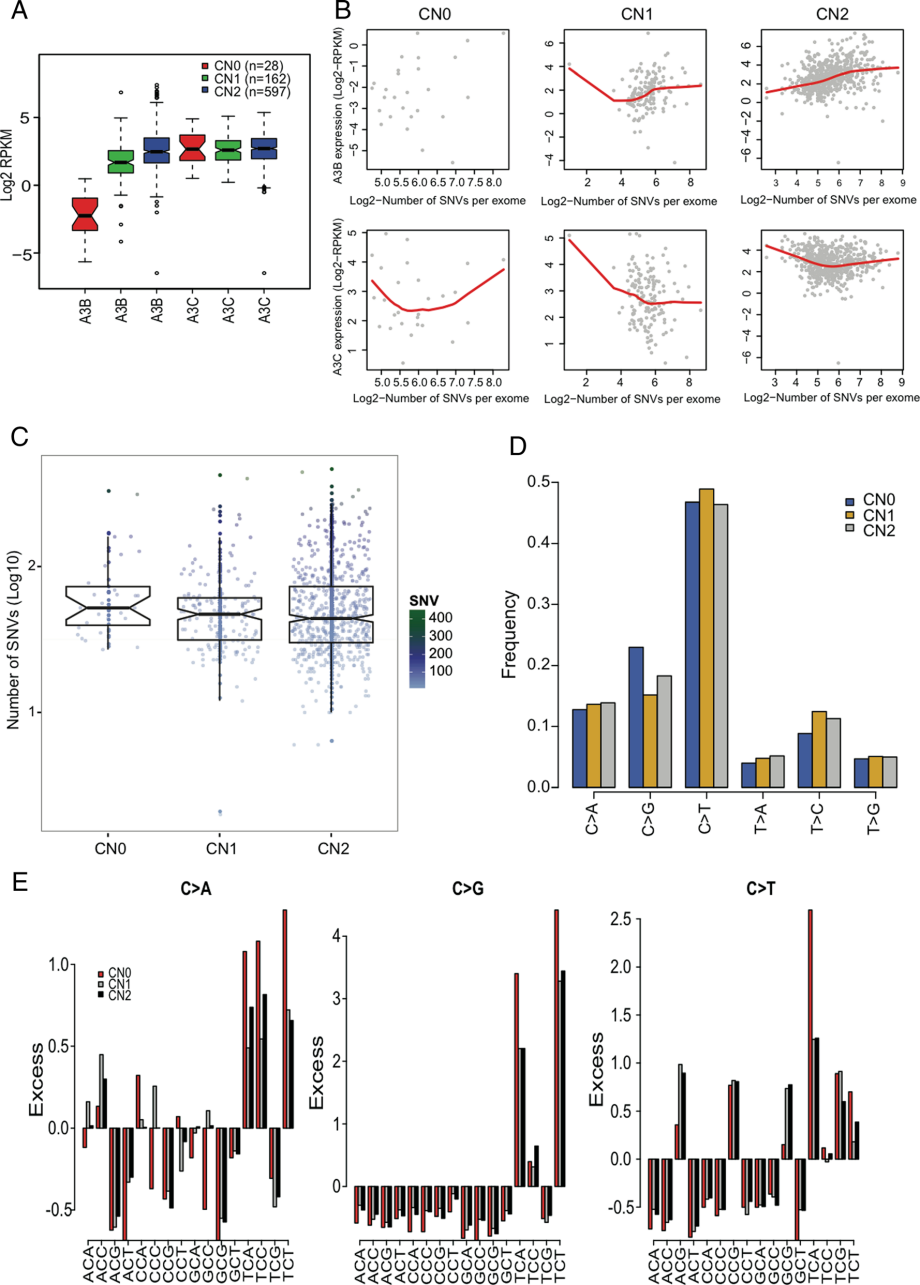
Gene expression and somatic mutation signatures in breast tumors stratified by copy numbers of *APOBEC3B* gene. **a** Boxplot of mRNA levels of *APOBEC3B* and *APOBEC3C* in totally 787 breast tumors. **b** Relationship between *APOBEC3B* (*upper panel*) and *APOBEC3C* (*lower panel*) expression levels and number of somatic SNVs per tumor exome. The *red curves* are drawn from the local regression smoothing. **c** Boxplot of the total number of somatic mutations per tumor exome (*y*-axis, log10 scaled). The *color bars* shown in the *right* are the original number of somatic SNVs per exome. **d** Frequency of each of six somatic mutation types. **e** Excess rate of trinucleotide motifs with centered C>A, C>G, and C>T mutations shown from *left* to *right panels*. The excess rate is calculated by (observed occurrence − mean occurrence) / mean occurrence. CN0, CN1, and CN2 present the zero, one, and two copies of *APOBEC3B* gene, respectively

In the analysis of the CNV status of *APOBEC3B* [30] in 787 breast tumor samples, we detected 190 breast cancer samples in carriers of germline deletion of *APOBEC3B* gene. Among them, 28 (75 % being ER+) and 162 (80 % being ER+) samples bear homozygous (zero copy, CN0) and heterozygous (one copy, CN1) deletions, respectively. Among the CN2 group (wild-type), 74 % of them are ER+. Figure 5a shows that the group with CN0 (homozygous deletion) effectively eliminates the transcription of *APOBEC3B*, and the CN1 group reduced mRNA level of *APOBEC3B*, compared to the CN2 group, indicating the high confidence of CNV calling in this study. We then examined the possibility of functional interaction between *APOBEC3B* and other APOBEC family members. In cancer samples with the depletion of *APOBEC3B*, although the expression level of the *APOBEC3A* is decreased, such change is likely a false positive as reported by Leonard et al. [13]. The mRNA levels for other APOBEC family members do not exhibit the remarkable alteration (Additional file 2: Figure S6), including the *APOBEC3C* gene (Fig. 5a), further raising the possibility that there are less functionally direct interactions between APOBEC family members, at least between *APOBEC3B* and other members.

To address whether the APOBEC-mediated mutagenesis in breast tumors is directly induced by the *APOBEC3B* protein, stratified by CNV of *APOBEC3B* locus, we further compared the number of mutations per tumor exome in breast cancer tissues. The results show the remarkably positive correlation (*r* = 0.33, *P* = 8.9 × 10^−16^) between the number of SNVs per exome and *APOBEC3B* expression levels for *APOBEC3B* with CN2, but not for CN1 and CN0 types (Fig. 5b). Similar but negative correlation of mutations per exome with *APOBEC3C* expression levels is observed. Meanwhile, the comparative results show that the number of somatic SNVs in the absence of *APOBEC3B* (CN0) is not significantly different with the other two genotypes (CN1 and CN2, Fig. 5c), although some biases could be introduced in the comparison, including the unequal sample size and regional (not whole-genome) variants for three genotypes. Furthermore, in terms of mutation types, the mutation patterns are quite similar with predominance of C-T transition for all three *APOBEC3B* genotypes (Fig. 5d). Compared with samples with CN1 and CN2 of *APOBEC3B*, we observed elevated frequency of C-G mutation in samples containing the CN0 of *APOBEC3B* (Fig. 5d). The excessive analysis of trinucleotide motifs with centered C>A, C>G, and C>T mutation types shows a significantly increased T*C*W motif occurrence in CN0 group (where W corresponds to A or T, Fig. 5e) compared with CN1 (*P* = 2.4 × 10^−4^) and CN2 groups (*P* < 2.2 × 10^−16^) based on Fisher’s exact test.

## Discussion

In this study, we investigated the genomic, transcriptomic, and epigenetic regulation of APOBEC gene family in breast cancers by integrating diverse high-throughput sequencing data. The transcriptome data from both breast cancer cell lines and tumor specimens reveal that the *APOBEC3B* and *APOBEC3C* genes show significant differences at the transcription levels between ER+ and ER− breast tumors. On the one hand, we recapitulated previous findings that the up-regulation of *APOBEC3B* gene represents a growing enzyme-catalyzed cytosine-to-uracil deamination activity in carcinogenesis [9, 10]. On the other hand, the substantial up-regulation of *APOBEC3B* gene and absence of down-regulation of *APOBEC3C* gene in ER− subtypes suggest that the cytosine deaminase activity for APOBECs may be distinct in cancer subtypes. Furthermore, our study also suggests that the APOBEC-induced cancer mutagenesis is distinct regarding ER status, in agreement with the phenomena of different mutational spectrum observed between ER+ and ER− breast cancers [15].

Our results provide an additional support of the functional significance of *APOBEC3B* and probably *APOBEC3C* in breast carcinogenesis [12]. Based on the overall similar mutation patterns between breast cancers in carriers of germline deletion of *APOBEC3B* genes and non-carriers, and a higher occurrence of somatic mutations on the T*C*W motif in *APOBEC3B* deletion samples, we speculate that germline deletion of *APOBEC3B*, expression of *APOBEC3B* and *APOBEC3C*, and somatic mutation may interact in contributing to breast cancer.

Integrated analysis of functional genomic data is a powerful approach with broad applications in many areas, including prediction of gene activity [31] and annotation of non-coding RNAs [32]. Using a similar approach, our integrated analyses illuminate the difference of chromatin states among APOBECs in breast cancer subtypes, which is highly consistent with the gene expression data. For instance, the active promoter and enhancer states at the *APOBEC3B* gene are concordant with the over-expression of *APOBEC3B*, particularly in ER− subtype. Meanwhile, the loss of active promoter and enhancer signals at the *APOBEC3C* gene in the ER+ cancer cell reflects the down-regulation of this gene observed in the ER+ subtype. In addition, the DNA hypermethylation may explain no or low expression profiles of other APOBECs. These results are concordant with Isobe’s report [33, 34] in blood cells. Meanwhile, Pauklin et al. [35] have reported the estrogen induced increase of *APOBEC3B* gene expression by nearly 1.5-fold in a dose-dependent manner, but not *APOBEC3C* gene and other members in human MCF-7 cells, suggesting the possibility that ER may directly (or indirectly) regulate APOBECs in breast cancer cells through recruiting chromatin modifiers [36].

Compared with *APOBEC3B*, the *APOBEC3C* gene may play a different role in the cancer genome mutagenesis. For example, we observed that the elevated expression levels of *APOBEC3C* but lowered expression levels of *APOBEC3B* in breast cancer patients have better clinical outcomes. The opposite correlation of *APOBEC3B* and *APOBEC3C* genes’ expression with clinical outcomes further provides a possibility of using both two genes as potential biomarkers in prognosis. Although no functional assays are performed in this study, several lines of evidence support the possible functionality of the APOBEC3C protein in breast cancer.

First, *APOBEC3C* is localized in both the cytoplasmic and nuclear compartments, which is not only observed in human cells [37–39], but also observed in the rhesus counterparts [7], suggesting functionally constrained localization of *APOBEC3C*. However, for other APOBECs, they show either tissue-restricted expression patterns (*APOBEC3A*, *APOBEC1*, *APOBEC2*, *APOBEC4*, and *AICDA*) or cytoplasmic localization (*APOBEC3D*, *APOBEC3F*, *APOBEC3G*, and *APOBEC3H*) in human cells [3, 4, 6, 7, 10, 39, 40]. Along with high expression levels of *APOBEC3C* gene in ER− breast cancer cells, it is possible that both *APOBEC3B* and *APOBEC3C* retain DNA cytosine deaminase activity in breast cancer. Meanwhile, Lackey et al. reported that throughout mitosis in HEK293T and HeLa cells, *APOBEC3C* has access to genomic DNA during interphase and telophase, while *APOBEC3B* is excluded from the genomic DNA during mitosis [37]. Such phenomena are consistent with our findings that there is less functional interaction between *APOBEC3C* and *APOBEC3B* proteins, suggesting they may perform different roles in cancer cells.

Second, given that the *APOBEC3C* possesses a single active Z2-cytosine deaminase domain, while the *APOBEC3B* has double Z-coordinating (Z1 and Z2) deaminase domains [8, 41], the crystal structure determinants and functional comparison have revealed the distinct substrate preferences for binding HIV-1 DNA between the single- and double-domained *APOBEC3* enzymes [42–44], raising the possibility that these two enzymes have differential DNA binding specificity which might help explain the relative differences in their observed mutagenesis in breast cancer cells, especially in the ER− cancers.

Third, besides the deaminase activity for APOBECs, the *APOBEC3* family proteins also contribute to inhibit the L1 retrotransposition in a deaminase independent manner [6, 38, 45]. For instance, depletion of *APOBEC3C* significantly increases the L1 retrotransposition activity by ~80 % in HeLa cells [39]. Meanwhile, many studies have strongly indicated the enhanced activity of LINE-1 retrotransposons in human cancers induces genome instability, DNA damage, and genetic variation [46–51], further implying the potentially functional roles of *APOBEC3C* in breast cancer.

## Conclusions

In conclusion, our integrated analyses suggested that *APOBEC3B* and *APOBEC3C* expression patterns were correlated with ER status and clinical outcome, providing an additional implication that these two genes may contribute to mutation profile and clinical outcome in breast cancer subtypes.

## Methods

### Data collection

We collected functional genomics data from experiments including the following: (1) RNA-seq, (2) chromatin immunoprecipitation followed by high-throughput DNA sequencing (ChIP-seq), and (3) bisulfite sequencing (BS-seq) data, for breast cell lines (including both normal and cancer) from the Gene Expression Omnibus (GEO) database [52] and ENCODE project. We initially searched the GEO database using the following terms: breast [AND] sequencing. After manual curation to rule out data where cell lines were treated by additional chemicals or siRNAs in experiments, all relevant data with no redundancy were retained for further analyses. We retrieved the ChIP-seq data released by the ENCODE project for well-characterized chromatin modifications in two breast cell lines: one normal cell line, called human mammary epithelial cells (HMEC), and the other ER+ breast cancer cell line (MCF-7). We also downloaded a total of 1000 RNA-seq data with approval (BAM files, Level 1) and corresponding clinical data (Biotab format) for breast tissue specimens (including 915 cancer and 85 adjacent normal tissue specimens) from TCGA (https://tcga-data.nci.nih.gov/tcga/). In addition, we downloaded 840 CEL files (Affymetrix SNP 6.0 array, level 1) and processed somatic mutation data (level 2) from 776 whole exome-sequencing (exome-seq) for the breast cancer from TCGA data portal, where 787 and 750 have matched RNA-seq data, respectively.

### Data processing and statistics

All raw sequencing data (FASTQ format) were initially mapped to the human reference genome (hg19) using Bowtie2 program [53] with the default setting. Aligned data in SAM format were processed and converted into BAM files using SAMtools program [54]. As each type of sequencing data conveys specifically biological purpose, we used the following methods to process the corresponding sequencing data. Except relevant programs described, all other bioinformatics analyses were implemented using Perl and R programming.

For RNA-seq data, we used similar methods described elsewhere [55]. In brief, to characterize the quantitative expression of each RefSeq gene, the reads per kilobase and million mapped reads (RPKM) was calculated as the number of mapped reads with the mapping quality (MAQ) ≥30 aligning to each transcript multiplied by 1 million and divided by the length of the transcript times the total number of aligned reads. Then the RPKM values for APOBEC family members were extracted to quantify their expression levels. The same method was also utilized to process BAM files for TCGA RNA-seq data.

To uniformly analyze and visualize ChIP-seq data, we used the MACS14 (version 1.4.2) algorithm [56] to call peaks of ChIP-seq data for histone modifications against the corresponding control data in 20 bp resolution (−−space = 20) at *P* < 1 × 10^−5^. The resulting peak files (.bed) were then implemented into the ChromHMM algorithm [57] at a 200-bp resolution for chromatin state characterization. We ran ChromHMM with a range of possible states and settled on a 9 state model as it accurately captured biologically meaningful patterns in a reproducible way. The resulting genome segmentation files were uploaded to the UCSC Genome Browser as a custom track to visualize the chromatin sates and comparison among APOBEC family members.

For BS-seq data, we employed the BSMAP (version 2.74) program [58] to analyze DNA methylation ratios on each CpG site. Each CpG site supported by ≥4 reads was considered. To compare the DNA methylation ratios among APOBECs, we calculated mean DNA methylation levels within each 100-bp sliding window across ±5 kb regions of transcription start site (TSS) for each of APOBEC family members.

Level 1 CEL files for the breast cancer were used for copy number variation (CNV) calling. CNVs were detected based on the signal intensities of over 1.8 million SNP or copy number probes on the Affymetrix SNP 6.0 array. The Affymetrix Power Tools (APT-1.14.3) package was used to normalize for samples run on the same plate according to file handles and the chemistry file available. CNVs were called using Birdsuite (version 1.4) [59], which calls both common and rare CNVs. The *APOBEC3B* deletion is named as CNP2576 (hg19, chr22: 39363620–39375307) in Canary and was determined by 24 probes. In the present study, we only focus on this *APOBEC3B* deletion and copy number states of 0, 1, and 2 (CN0, CN1, and CN2) were used. Genotypes were checked against sample barcodes and participant IDs to identify possible duplicate samples.

For somatic mutation data, both somatic single nucleotide variants (SNVs) and C>T/G>A mutations per tumor were first summed. The correlation between the number of C>T/G>A mutations (or somatic SNVs) and expression levels of APOBECs was calculated using non-parametric Spearman’s Rank correlation statistics. The same analysis was employed stratifying for *APOBEC3B* germline deletion states. Six types of somatic SNVs (C>A/G>T, C>G/G>C, C>T/G>A, T>A/A>T, T>C/A>G, T>G/A>C) were counted and their relative frequencies were then calculated and compared. Following the method reported in the previous study [10], we compared the trinucleotides motif patterns with the background distribution normalized among breast cancer samples in different copies of *APOBEC3B* gene.

## Additional files

Additional file 1:
**Tables S1 and S2.** Table S1. Data characteristics in this study. Table S2. Expression levels of APOBEC family genes across ten breast cell lines. (XLSX 14 kb) Additional file 2: Figures S1–S6. Figure S1. Expression profiles of APOBEC family genes in breast tumor tissues and adjacent normal tissues in relation to ER status. Figure S2. Comparison of chromatin states in *APOBEC1* (A), *APOBEC2* (B), *APOBEC4* (C), and *AICDA* (D) genes in ER+ (MCF-7), ER− (HCC1954) breast cancer cells, and normal cells (HMEC). Chromatin states characterized by the ChromHMM algorithm are represented by different colors shown in the bottom. Figure S3. Kaplan-Meier curve for overall survival of four patient groups with higher (top 50 %) or lower (bottom 50 %) expression of *APOBEC3B* and *APOBEC3C* genes in breast cancer. Figure S4. Distribution of the number of somatic mutations (A) and C>T/G>A mutations (B) per tumor exome. The red curve is a kernel density estimate. Figure S5. Relationship between mRNA levels of *APOBEC3B* (upper panel) and *APOBEC3C* (lower panel) and number of somatic SNVs per tumor exome stratified by the ER status. The black lines and red curves are drawn from the linear regression model and local regression smoothing, respectively. Figure S6. Expression profiles of APOBEC family genes in breast tumor tissues in relation with copy numbers of *APOBEC3B*. (ZIP 559 kb)
