# Supplementary figures and images for: Integrative genomic analysis reveals functional diversification of APOBEC gene family in breast cancer
Source: Hum Genomics. 2015 Dec 18;9:34. doi: 10.1186/s40246-015-0056-9 (PMC4684623; doi:10.1186/s40246-015-0056-9)

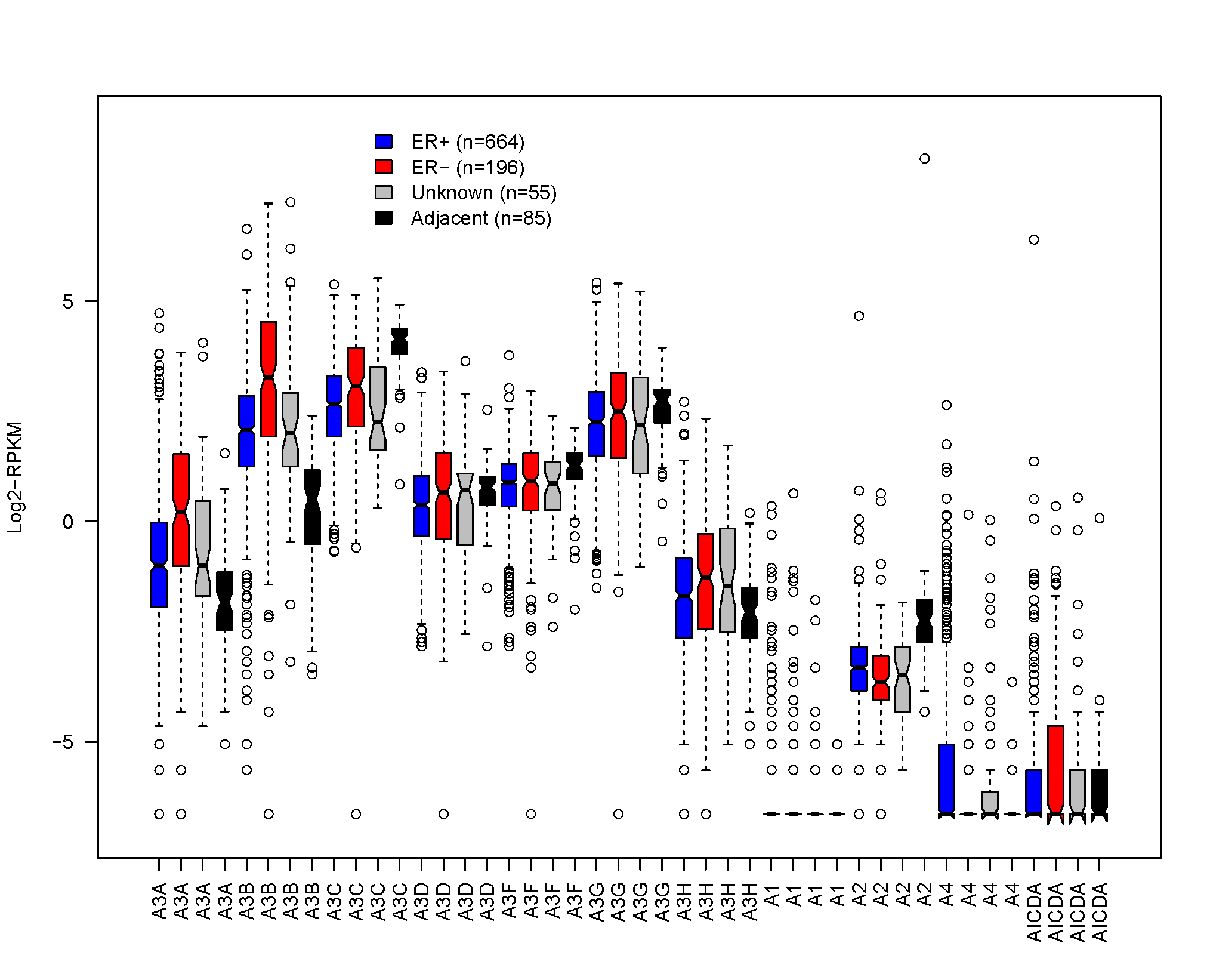

Supplement: Additional file 2: Figures S1–S6. — Figure S1. Expression profiles of APOBEC family genes in breast tumor tissues and adjacent normal tissues in relation to ER status. Figure S2. Comparison of chromatin states in APOBEC1 (A), APOBEC2 (B), APOBEC4 (C), and AICDA (D) genes in ER+ (MCF-7), ER− (HCC1954) breast cancer cells, and normal cells (HMEC). Chromatin states characterized by the ChromHMM algorithm are represented by different colors shown in the bottom. Figure S3. Kaplan-Meier curve for overall survival of four patient groups with higher (top 50 %) or lower (bottom 50 %) expression of APOBEC3B and APOBEC3C genes in breast cancer. Figure S4. Distribution of the number of somatic mutations (A) and C>T/G>A mutations (B) per tumor exome. The red curve is a kernel density estimate. Figure S5. Relationship between mRNA levels of APOBEC3B (upper panel) and APOBEC3C (lower panel) and number of somatic SNVs per tumor exome stratified by the ER status. The black lines and red curves are drawn from the linear regression model and local regression smoothing, respectively. Figure S6. Expression profiles of APOBEC family genes in breast tumor tissues in relation with copy numbers of APOBEC3B. (ZIP 559 kb) [file 40246_2015_56_MOESM2_ESM.zip › Figure S1.tiff]

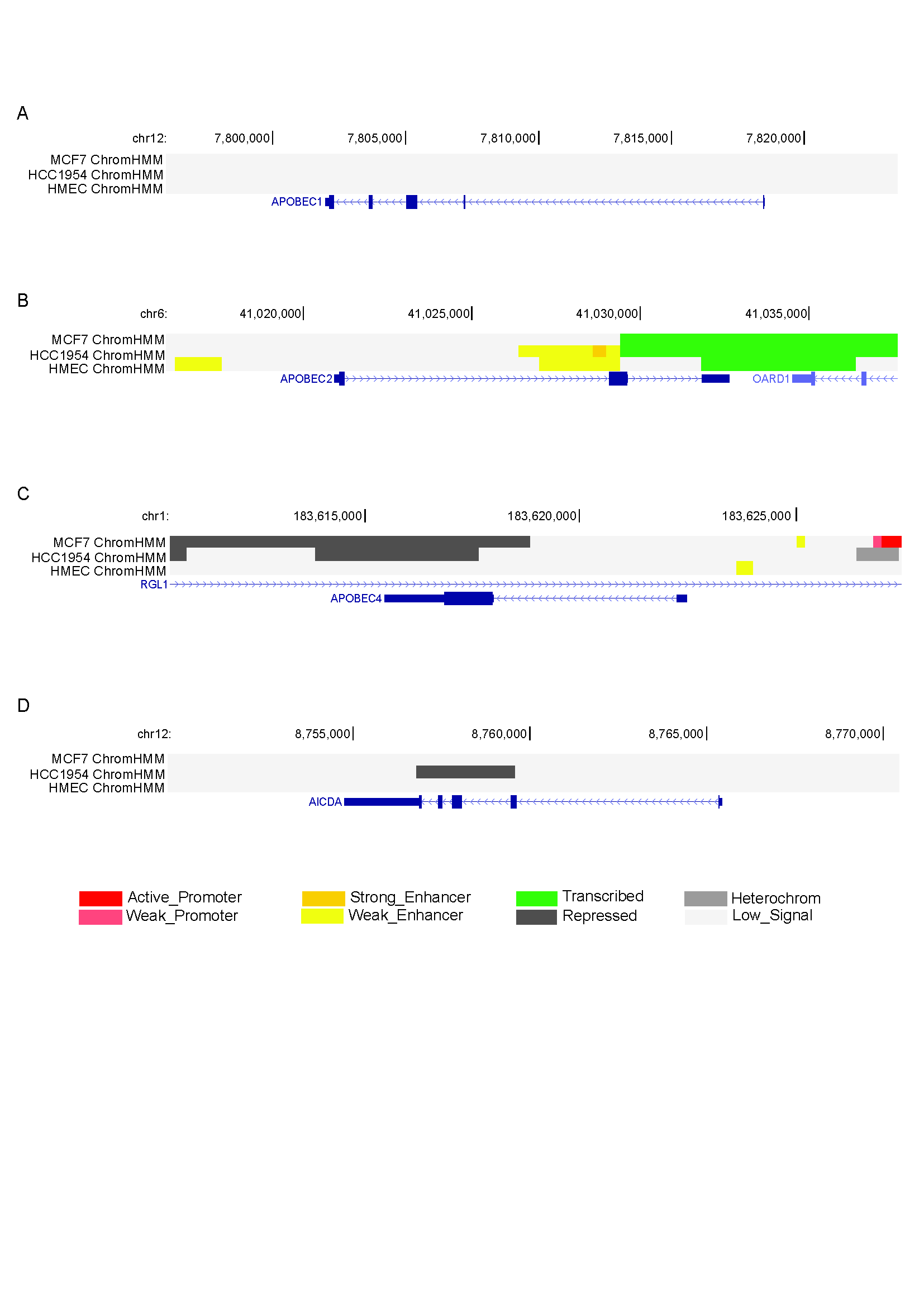

Supplement: Additional file 2: Figures S1–S6. — Figure S1. Expression profiles of APOBEC family genes in breast tumor tissues and adjacent normal tissues in relation to ER status. Figure S2. Comparison of chromatin states in APOBEC1 (A), APOBEC2 (B), APOBEC4 (C), and AICDA (D) genes in ER+ (MCF-7), ER− (HCC1954) breast cancer cells, and normal cells (HMEC). Chromatin states characterized by the ChromHMM algorithm are represented by different colors shown in the bottom. Figure S3. Kaplan-Meier curve for overall survival of four patient groups with higher (top 50 %) or lower (bottom 50 %) expression of APOBEC3B and APOBEC3C genes in breast cancer. Figure S4. Distribution of the number of somatic mutations (A) and C>T/G>A mutations (B) per tumor exome. The red curve is a kernel density estimate. Figure S5. Relationship between mRNA levels of APOBEC3B (upper panel) and APOBEC3C (lower panel) and number of somatic SNVs per tumor exome stratified by the ER status. The black lines and red curves are drawn from the linear regression model and local regression smoothing, respectively. Figure S6. Expression profiles of APOBEC family genes in breast tumor tissues in relation with copy numbers of APOBEC3B. (ZIP 559 kb) [file 40246_2015_56_MOESM2_ESM.zip › Figure S2.tiff]

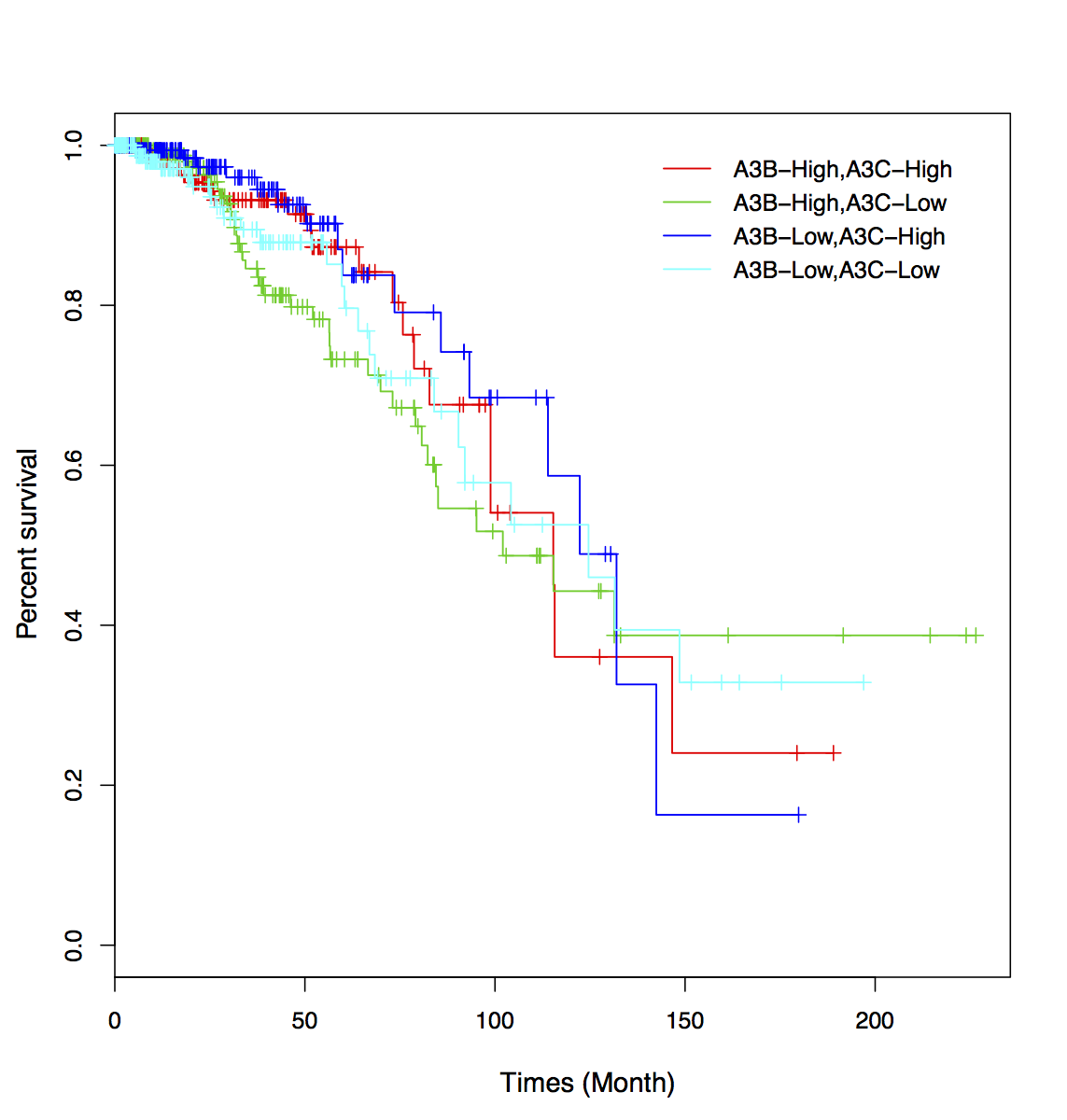

Supplement: Additional file 2: Figures S1–S6. — Figure S1. Expression profiles of APOBEC family genes in breast tumor tissues and adjacent normal tissues in relation to ER status. Figure S2. Comparison of chromatin states in APOBEC1 (A), APOBEC2 (B), APOBEC4 (C), and AICDA (D) genes in ER+ (MCF-7), ER− (HCC1954) breast cancer cells, and normal cells (HMEC). Chromatin states characterized by the ChromHMM algorithm are represented by different colors shown in the bottom. Figure S3. Kaplan-Meier curve for overall survival of four patient groups with higher (top 50 %) or lower (bottom 50 %) expression of APOBEC3B and APOBEC3C genes in breast cancer. Figure S4. Distribution of the number of somatic mutations (A) and C>T/G>A mutations (B) per tumor exome. The red curve is a kernel density estimate. Figure S5. Relationship between mRNA levels of APOBEC3B (upper panel) and APOBEC3C (lower panel) and number of somatic SNVs per tumor exome stratified by the ER status. The black lines and red curves are drawn from the linear regression model and local regression smoothing, respectively. Figure S6. Expression profiles of APOBEC family genes in breast tumor tissues in relation with copy numbers of APOBEC3B. (ZIP 559 kb) [file 40246_2015_56_MOESM2_ESM.zip › Figure S3.tiff]

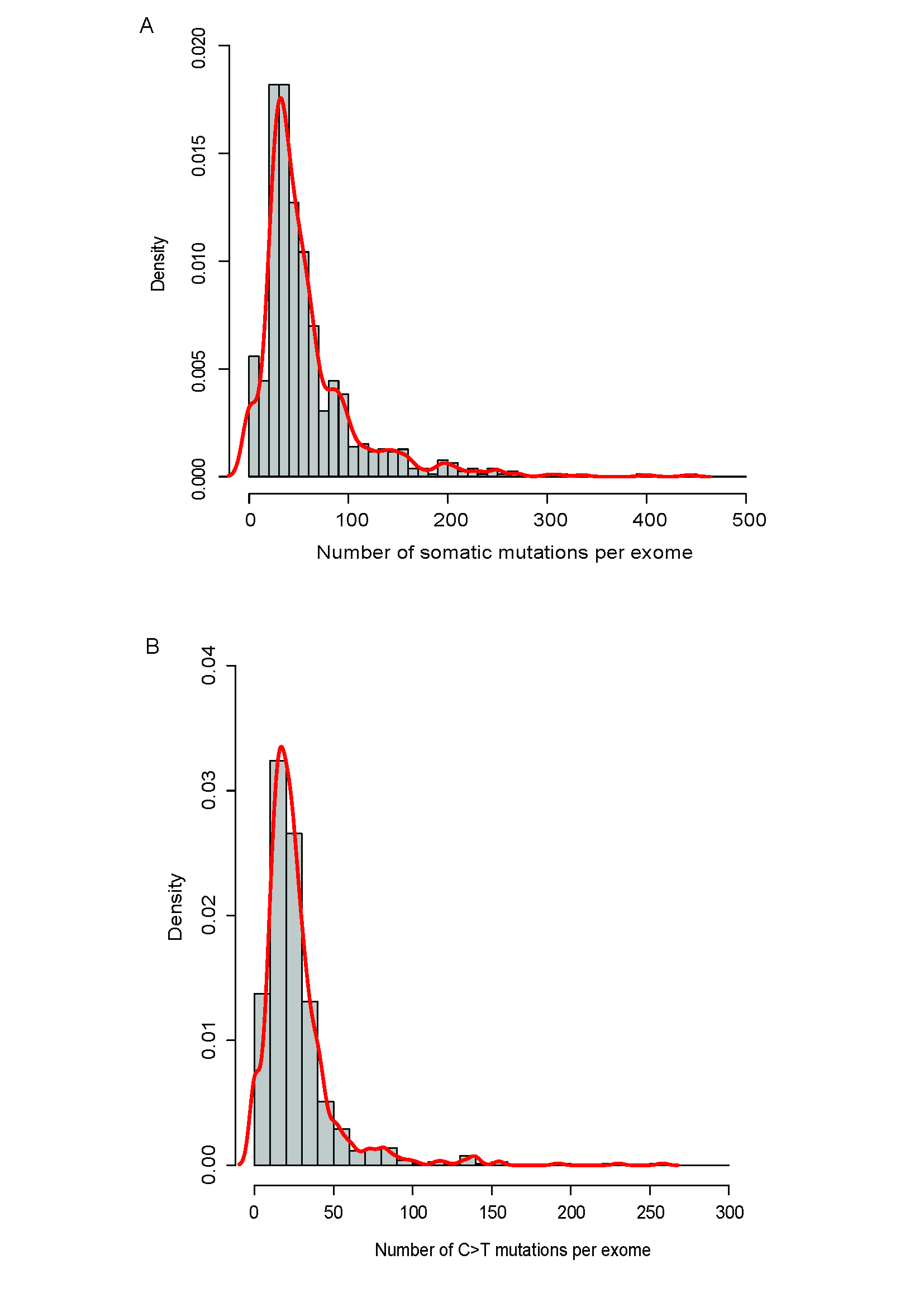

Supplement: Additional file 2: Figures S1–S6. — Figure S1. Expression profiles of APOBEC family genes in breast tumor tissues and adjacent normal tissues in relation to ER status. Figure S2. Comparison of chromatin states in APOBEC1 (A), APOBEC2 (B), APOBEC4 (C), and AICDA (D) genes in ER+ (MCF-7), ER− (HCC1954) breast cancer cells, and normal cells (HMEC). Chromatin states characterized by the ChromHMM algorithm are represented by different colors shown in the bottom. Figure S3. Kaplan-Meier curve for overall survival of four patient groups with higher (top 50 %) or lower (bottom 50 %) expression of APOBEC3B and APOBEC3C genes in breast cancer. Figure S4. Distribution of the number of somatic mutations (A) and C>T/G>A mutations (B) per tumor exome. The red curve is a kernel density estimate. Figure S5. Relationship between mRNA levels of APOBEC3B (upper panel) and APOBEC3C (lower panel) and number of somatic SNVs per tumor exome stratified by the ER status. The black lines and red curves are drawn from the linear regression model and local regression smoothing, respectively. Figure S6. Expression profiles of APOBEC family genes in breast tumor tissues in relation with copy numbers of APOBEC3B. (ZIP 559 kb) [file 40246_2015_56_MOESM2_ESM.zip › Figure S4.tiff]

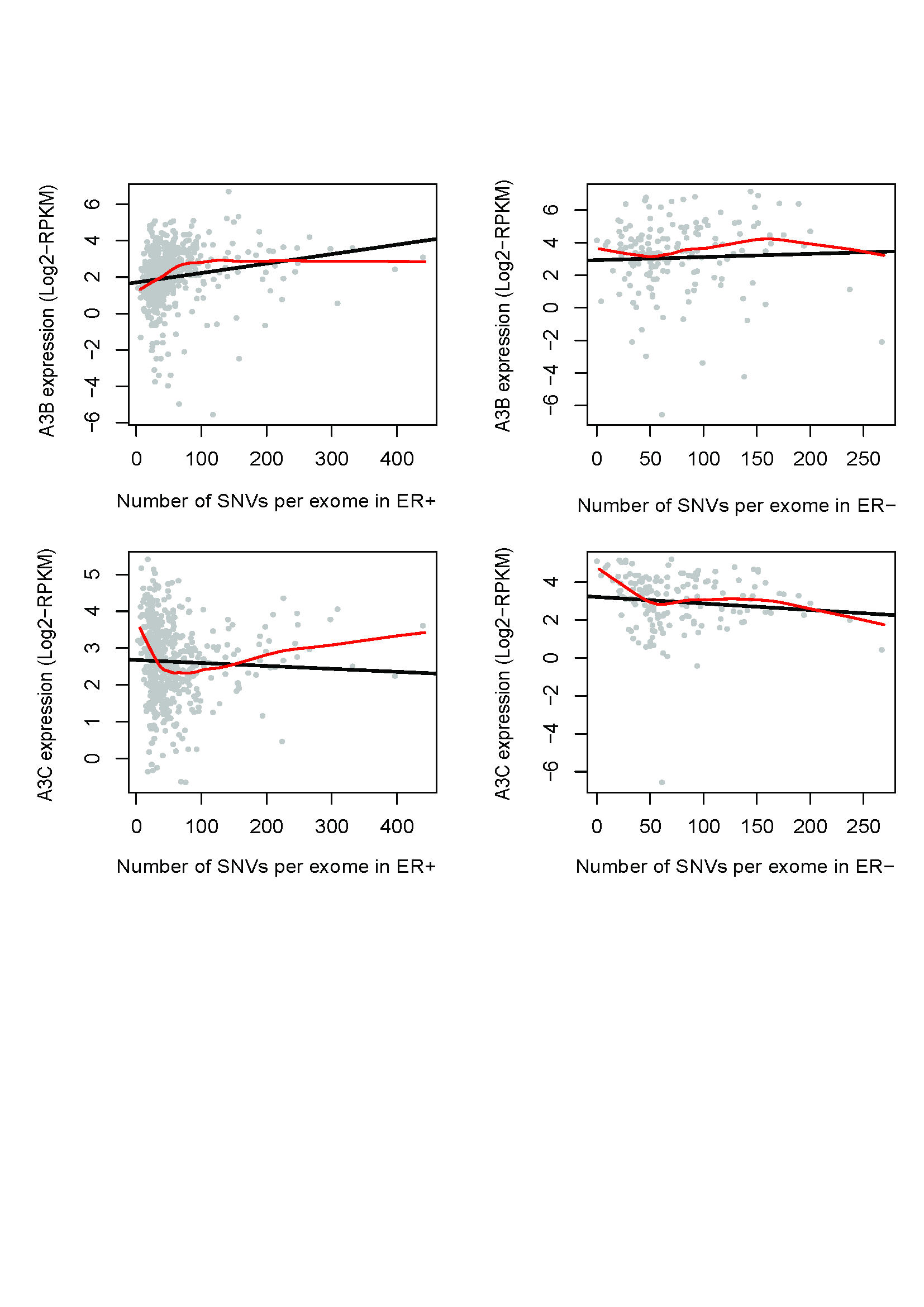

Supplement: Additional file 2: Figures S1–S6. — Figure S1. Expression profiles of APOBEC family genes in breast tumor tissues and adjacent normal tissues in relation to ER status. Figure S2. Comparison of chromatin states in APOBEC1 (A), APOBEC2 (B), APOBEC4 (C), and AICDA (D) genes in ER+ (MCF-7), ER− (HCC1954) breast cancer cells, and normal cells (HMEC). Chromatin states characterized by the ChromHMM algorithm are represented by different colors shown in the bottom. Figure S3. Kaplan-Meier curve for overall survival of four patient groups with higher (top 50 %) or lower (bottom 50 %) expression of APOBEC3B and APOBEC3C genes in breast cancer. Figure S4. Distribution of the number of somatic mutations (A) and C>T/G>A mutations (B) per tumor exome. The red curve is a kernel density estimate. Figure S5. Relationship between mRNA levels of APOBEC3B (upper panel) and APOBEC3C (lower panel) and number of somatic SNVs per tumor exome stratified by the ER status. The black lines and red curves are drawn from the linear regression model and local regression smoothing, respectively. Figure S6. Expression profiles of APOBEC family genes in breast tumor tissues in relation with copy numbers of APOBEC3B. (ZIP 559 kb) [file 40246_2015_56_MOESM2_ESM.zip › Figure S5.tiff]

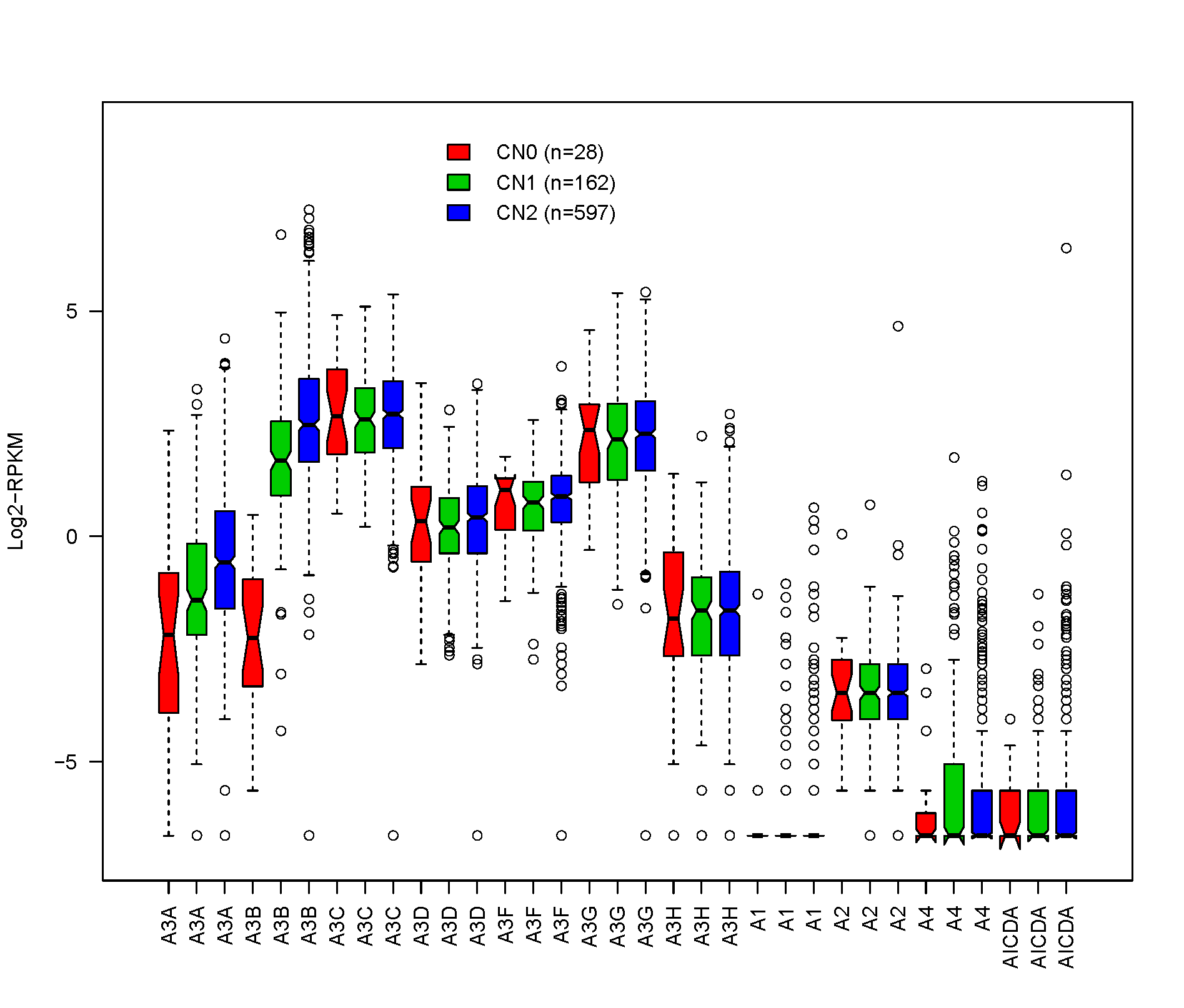

Supplement: Additional file 2: Figures S1–S6. — Figure S1. Expression profiles of APOBEC family genes in breast tumor tissues and adjacent normal tissues in relation to ER status. Figure S2. Comparison of chromatin states in APOBEC1 (A), APOBEC2 (B), APOBEC4 (C), and AICDA (D) genes in ER+ (MCF-7), ER− (HCC1954) breast cancer cells, and normal cells (HMEC). Chromatin states characterized by the ChromHMM algorithm are represented by different colors shown in the bottom. Figure S3. Kaplan-Meier curve for overall survival of four patient groups with higher (top 50 %) or lower (bottom 50 %) expression of APOBEC3B and APOBEC3C genes in breast cancer. Figure S4. Distribution of the number of somatic mutations (A) and C>T/G>A mutations (B) per tumor exome. The red curve is a kernel density estimate. Figure S5. Relationship between mRNA levels of APOBEC3B (upper panel) and APOBEC3C (lower panel) and number of somatic SNVs per tumor exome stratified by the ER status. The black lines and red curves are drawn from the linear regression model and local regression smoothing, respectively. Figure S6. Expression profiles of APOBEC family genes in breast tumor tissues in relation with copy numbers of APOBEC3B. (ZIP 559 kb) [file 40246_2015_56_MOESM2_ESM.zip › Figure S6.tiff]
